# News and Notes

**Published:** 1908-01

**Authors:** 


### NEWS AND NOTES.

The health officer of Richmond has arranged for the opening at an early date of two tuberculosis dispensaries.

Dr. John H. Musser, of the medical department of the University of Pensylvania, was the guest of honor at a banquet of the Alpha Omega Fraternity at the St. Louis Club, on Friday evening, November 8th.

The North Carolina Hospital Commission proposes to spend $250,000 annually for the next four years in enlarging the state hospitals for the insane.

In the first week in October, the new German Urologic Society, founded at Stuttgart, last year, held its first congress in Vienna.

At the commitment trial, Dr. Elmore was freed, as the judge held that the killing was justifiable.

The Sixth District Medical Association of Georgia held a successful meeting at Macon on Nov 13th with the following program:

Address of Welcome; Hon. Bridges Smith. Response by Dr. A.F.White, Flovilla. "A Successful case of Laminectomy," by Dr. H.J.Williams, Macon. "Cephalocele," by Dr. J.A.Combs, Locust Grove. "Pemphigust Neonatorum," by Dr. I.H.Adams, Macon. "Complications of Measles; their prevention and treatment," by Dr. C.C.Harrold, Macon. "Amenorrhoea," by Dr. J.A.Jarrell, Jackson. "Sixth District Medical Association and its Mission," by Dr. J.R.Shannon, Cabaniss. "Medicinal effect of Indian Springs Water in the treatment of Typhoid Fever," by Dr. A.F.White, Flovilla.

---

Dr. Kenneth W. Millican was the guest of honor at a farewell banquet given by his medical friends of St. Louis, November 8.

---

A number of Vienna physicians have organized to facilitate the renting of automobiles by physicians and to reduce garage and chauffeur expenses so that the total expense will be no more than physicians have been in the habit of bearing for other rented conveyances.

---

The colored physicians of Indianapolis, October 16 organized a society known as the Æsculapian Society, to meet once a month having for its the object the education of the colored race along proper hygienic lines.

---

It is stated that arrangements have been made for the erection of a fireproof hospital in Atlantic City for the treatment of contagious diseases. The institution will comprise three buildings and will cost about $92,000.

---

At the instance of the State Board of Examiners of Utah, the attorney of Salt Lake County on October 9 commenced an action to revoke the license issued to Earl S. Beers, "the boy phenomenal." The court is also asked to issue a perpetual injunction against this individual practicing medicine in the state.

At the Columbus, O., meeting of the Mississippi Valley Medical Association, the following officers were elected: President, Arthus R. Elliott, Chicago; first vice-president, F.FLawrence, Columbus; second vice-president, R.C.McCord, Lebanon, Ky; secretary, Henry Enos Tuley, Louisville, Ky; treasurer, S.C. Stanton, Chicago. Louisville was chosen for the next meeting place.

---

The calendar of the Royal College of Surgeons has just been published by the council. It shows that there are 1,387 fellows on the roll of the college. There are 17,544 members and 398 licentiates in midwifery. The licentiates in dental surgery are stated to number 2,111, holders of the diploma of public health granted by the Royal College of Physicians and Surgeons number 601. The income from all sources amounted to $121,500 and the expenses for the year to $113,000.

---

DR. W. E. CAMPBELL and DR. R. B. RIDLEY, JR., have formed a partnership under the name of Drs. Campbell and Ridley. They will have their offices at 605-6-7-8- Century Building. They will continue to limit their work to diseases of the eye, ear, nose and throat, as they have done in the past.

---

Dr. B. V. Elmore, of Kestler, Ga., shot and killed Jeff Spires of Miller Co., Nov 29th in defense of his father, R.E. Elmore, whose life was threatened by Spires. The occasion of the shooting was a feud of a years standing between Elmore and Spires; the latter, while drinking, drew a knife and placed it at R.E. Elmore's throat, declaring his intention of killing him. Before the threat could be executed, young Dr. Elmore, who graduated last year from the Atlanta School of Medicine, fired and killed Spires.

---

According to the report of the New York State Commission in Lunacy, the total number of insane patients in New York institutions for the year ending September 30, 1906, was 26,357, or about one for every 300 of the population. The report shows that insanity has been steadily increasing in the State since the year 1897, and that insanity is more prevalent among persons of foreign birth than among natives.

---

The Honolulu Board of Health is said to have decided to permit J. Lor Wallach, who claims that he can cure leprosy, to treat the lepers at the Molokai settlement.

---

The Chattahoochee Valley Medical and Surgical Association, which was organized this year, will hold its second meeting in Columbus, January 14-16.

---

Dr. James E. Sullivan has been nominated by the democrats of the town of Narragansett, Rhode Island, for State senator.

---

The Ladies' Ambulance Association, Greensboro, has presented St. Leo's Hospital, in that city, witih an ambulance which cost $800.

---

On November 4th, Dr. Carlos Finlay, chief of the Department of Health and Sanitation of Havana, was presented by Governor Magoon with the Mary Kingsley medal in recognition of his work in connection with the transmission of yellow fever by mosquitoes.

---

Reports from health officers show that there are over fifty undoubted cases of plague in San Francisco and about thirty suspected cases under observation. Twenty deaths have been recorded so far.

---

Dr. F.P.Calhoun, son of Dr. A.W.Calhoun, was elected to the medical staff at the monthly meeting of the Grady hospital trustees to fill the vacancy made by the resignation of his father, which was received a short time ago.

Recorder Broyles imposed a fine of $50.75 on J.H. Nelms, an East Atlanta dairyman, for selling watered milk with too small a percentage of fat. The recent rigid laws have greatly improved the quality of the milk, and Dr. Claude Smith, the City Bacteriologist, claims that the infant mortality during the summer months was reduced about 13 per cent.

---

Judge Kavanaugh, of Chicago, has collected statistics which show that there have been 43,000 homicides in the United States within the last five years; this number is 8,000 more than were killed by gun shot wounds in the civil war.

---

Dr. Littell Funkhauser, of Rome, Ga., was recently injured by a runaway horse driven by a rural mail carrier; the horse ran into the doctor's buggy, threw him out and wrecked both buggies.

---

Dr. C. H. Waddell Styles, of the U.S. Public Health and Marine Hospital Service, was recently in Atlanta and delivered interesting lectures at the Atlanta College of Physicians and Surgeons and at the Atlanta School of Medicine.

Dr. Styles' chief theme was the "Economic Side of Hookworm Disease;" this subject is one in which he is particularly well versed and undoubtedly much good will follow the dissemination of facts regarding the importance of this disease from an economic standpoint.

---

According to an editorial in the last issue of Northwest Medicine, considerable apprehension is felt over the appearance of the plague in Seattle, and, although the daily press has minimized the dangers of the situation, it is claimed that when it gains a foothold in a certain section, it is there to stay for a long period of years, in spite of all the hygienic and sanitary measures.

---

Fines of $50 and costs for violation of the cocaine ordinance were imposed against three druggists—J.C. Coursey, of the Whitaker-Coursey Drug Co., A.R. Munn, clerk for the same company, and A.C. Wessell, of Jackson and Wessell, 28 Marietta St.

Dr. J.H. King, a practicing physician, was held for the state court in two cases for violating the state law regulating the sale of cocaine and other narcotic drugs.

——o——

Medical officers of the United States Army will hereafter make a strict examination of all soldiers who have been ordered to Cuba or the Philippine Islands, and any who are found to be afflicted with infectious or contagious diseases will be reported for immediate discharge or transfer to commands stationed within the United States. All troops ordered to take transports, either to Cuba or the Philippines will require to show certificates of medical inspection and vaccination.

——o——

In the annual report of the chief of the Bureau of Medicine and Surgery to the Secretary of the Navy, he states that great difficulty is experienced in recruiting the medical corps to its normal strength. The conditions which tend to bring about this difficulty are a disproportion of the grades, a sluggish promotion and deprivation of titles.

——o——

At the meeting of the faculty of the Louisville Medical College and the Hospital College of Medicine, October 18, the two faculties were combined under the name of the Louisville and Hospital Medical College, continuing as the medical department of Central University. The faculties of the joint institutions will consist of the same men as those heretofore composed the faculties of the separate institutions.

——o——

Dr. J.J. Lawrence has sold the Medical Brief, St. Louis, to Mr. Henry R. Strong.

——o——

According to the semi-annual report of the State Board of Health, the death rate of Oregon is only eight per thousand.

——o——

Arrangements are well under way for the establishment in Augusta of a public hospital, at a cost of not less than $50,000.

### GRADY HOSPITAL.

The Medical Board of the Grady Hospital accepted the report of the special Committee appointed to investigate the recent charges of harsh treatment and over-work of the nurses, without further investigation. It was found that there was not sufficient grounds for the complaint of the under-graduate nurses, the trouble being nothing more than chafing under the necessary discipline and training. They determined, however, to enlarge the nursing staff of the hospital and to equalize the work of the head nurses by shifting them at given intervals from one ward to another.

A bureau of information to give out authoritative reports is to be established in the near future.

Especial attention is called to the inadequate quarters now occupied by the nurses and the council is to be urged to furnish a more comfortable and attractive home for them.

### INTERNATIONAL CONGRESS OF TUBERCULOSIS.

Progress along all lines connected with the International Congress on Tuberculosis which is to take place in Washington from Sept. 21 to Oct. 12, 1908, was shown by the reports presented at a meeting of the Committee of Arrangements, held in New York, at the Associated Charities Building, Monday evening, Oct. 28. Dr. Lawrence F. Flick, of Philadelphia, Chairman of the Committee, presided, and the other members present were Dr. Joseph Walsh, Philadelphia, Secretary, Dr. John S. Fulton, Washington, Secretary-General, Mr. William H. Baldwin, Washington, Dr. Herman M. Biggs, New York, Dr. Frank Billings, Chicago, Mr. Edward T. Devine, New York, Mr. Livingston Farrand, New York, Dr. J.C. Greenway, Greenwich, Conn., Dr. Chas. J. Hatfield, Philadelphia, Dr. Abraham Jacobi, New York, Dr. Alfred Meyer, Mrs. James. E. Newcomb, New York, Gen. Geo. M. Sternberg, Washington and Dr. Wm. H. Welch, Baltimore.

The meeting was the first held since Dr. Flick's return from abroad, and his reports of his visits to the International Conference on Tuberculosis in Vienna and to the International congress on Hygiene and Demography, at Berlin, were interesting features of the session. More than a thousand delegates were registered at Vienna, he said, and the gathering at Berlin was quite as large. The leading men in both associations are looking forward with a great deal of enthusiasm, Dr. Flick said, to the meeting in Washington next year, and about four hundred of the members of the foreign organizations may be expected to attend the Congress. The Conference selected this country as its place of meeting in 1908, just as the Congress did two years ago. The Conference and the Congress are two distinct organizations. The International Conference on Tuberculosis meets every year and keeps up a continuous organization with hearquarters in Berlin. The International Congress on Tuberculosis meets only once in three years and does not maintain an international bureau in the intervals. Dr. Flick stated that at the International Conference, interest centered especially in the time-worn subject of the routes of invasion for the tubercle baccillus. It seems to be demonstrated that the disease may be contracted by both the respiratory route and the alimentary route. Though this does not make us much wiser in a practical way, still it is somewhat comforting to know that the respiratory route is less important than it was once thought to be. On the other hand, that information is compensated by the importance of the alimentary route.

In connection with his account of the progress made in the preliminary arrangements for the International Congress on Tuberculosis, Dr. John S. Fulton, the Secretary-General, reported that ten distinguished foreigners have consented to participate in the series of special addresses that are to form a part of the program. The names of these eminent specialists follow:— Dr. R.W. Philip, Edinburgh; Dr. Theodore Williams, London; Dr. Arthur Newsholme, Health Officer, Brighton, England; Dr. C.H. Spronck Utrecht, Holland; Dr. Karl Turban, Davos-Platz, Switzerland; Dr. Gotthold Pannwitz, Charlottenburg; Dr. Emil von Behring, Marburg; Dr. A. Calmette, Pasteur Institute, Lisle, France; Dr. Maurice Letulle, Paris; and Dr. S. Kitasato, Tokyo, Japan.

Dr. Fulton also reported that up to the date of the meeting, the Governors of twenty-three states had lent official auspices to the Congress. This not only insures official representation so far as that many states are concerned, but it insures an active organization in each of these states, that will be interested in the

Congress. The states in which this action has been taken so far, are

| | |
|---|---|
| California, | Kansas, |
| Utah, | Tennessee, |
| Montana, | South Carolina, |
| North Dakota, | North Carolina, |
| Minnesota, | Maryland, |
| Wisconsin, | New York, |
| Illinois, | Massachusetts, |
| Iowa, | Vermont, |
| Indiana, | Maine, |
| Michigan, | West Virginia, |
| Ohio, | Missouri, |
| Kentucky. | |

Reporting on the formation of State Committees, the Secretary-General said that such committees had been appointed in nearly all of the states in the United States; that several have already organized and are earnestly at work. He reported also that replies have been received from various foreign countries in reference to the appointment of committees, and the replies indicate that the countries addressed will be represented in nearly every instance by exhibits, as well as by delegates.

